# Apoptosome formation upon overexpression of native and truncated Apaf-1 in cell-free and cell-based systems

**DOI:** 10.1016/j.abb.2018.01.017

**Published:** 2018-03-15

**Authors:** Ali Reza Noori, Elaheh Sadat Hosseini, Maryam Nikkhah, Saman Hosseinkhani

**Affiliations:** a Department of Biochemistry, Faculty of Biological Sciences, Tarbiat Modares University, Tehran, Iran; b Department of Nanobiotechnology, Faculty of Biological Sciences, Tarbiat Modares University, Tehran, Iran

**Keywords:** Apoptosis, Apaf-1, Split-luciferase, Truncated Apaf-1, Cell death, Apoptosome, Cell-free system

## Abstract

Apaf-1 is a cytosolic multi-domain protein in the apoptosis regulatory network. When cytochrome *c* releases from mitochondria; it binds to WD-40 repeats of Apaf-1 molecule and induces oligomerization of Apaf-1. Here in, a split luciferase assay was used to compare apoptosome formation in cell-free and cell-based systems. This assay uses Apaf-1 tagged with either N-terminal fragment or C-terminal fragment of *P. pyralis* luciferase. In cell based-system, the apoptosome formation is induced inside the cells which express Apaf-1 tagged with complementary fragments of luciferase while in cell-free system, the apoptosome formation is induced in extracts of the cells. In cell-free system, cytochrome *c* dependent luciferase activity was observed with full length Apaf-1. However, luciferase activity due to apoptosome formation was much higher in cell based system compared to cell-free system. The truncated Apaf-1 which lacks WD-40 repeats (ΔApaf-1) interacted with endogenous Apaf-1 in a different fashion compared to native form as confirmed by different retention time of eluate in gel filtration and binding to affinity column. The interactions between endogenous Apaf-1 and ΔApaf-1 is stronger than its interaction with native exogenous Apaf-1 as indicated by dominant negative effect of ΔApaf-1 on caspase-3 processing.

## 1 Introduction

Apoptosis is a highly organized pathway, an essential process for maintaining the physiological balance between death and cell growth. Disruption of this process is involved in many pathological conditions such as Alzheimer disease, ischemia and autoimmune disorders. However, this process is needed for embryonic development and immune system function [[1], [2], [3], [4]]. Apaf-1 is an adaptor molecule in formation of apoptosome heptameric complex. When cytochrome *c* is released from mitochondria, it binds to Apaf-1 and nucleotide exchange occurs; this lead to Apaf-1 activation and oligomerization. Caspase-9, a key caspase in the mitochondrial cell death pathway [5,6] is then activated by the oligomeric Apaf-1. Apaf-1 is a multi-domain protein, including caspase recruitment domain (CARD) that interacts with procaspase-9, a central domain which involves in nucleotide-binding and oligomerization domain (NOD) and C-terminal multiple WD-40 repeats that are suggested to play a regulatory role in Apaf-1 function [[7], [8], [9], [10]]. The released cytochrome *c* binds to WD-40 repeats of Apaf-1, being in a locked autoinhibited form [9], and exerts structural changes in Apaf-1, leading to exposure of nucleotide binding sites to dATP/ATP. By hydrolysis of the bound (d)ATP and (d)ADP exchange, Apaf-1 oligomerization occurs and the wheel like signaling complex forms [2,7]. So far, two models have been proposed to explain the Apaf-1: Apaf-1 interactions [11,12]. CARD: CARD interactions provides a platform for Caspase-9 binding but other interactions are required and this binding triggers caspase-9 activation [13,14]. The presence of caspase-9 on the apoptosome complex caused more efficient cleavage of procaspase-3 that cause apoptosis [2].

According to previous reports, removal of C-terminus WD-40 domain leads to the formation of mini-apoptosome even in the absence of cytochrome *c* [15]. This apoptosome could activate caspase-9, but is unable to activate caspase-3 [16]. These results indicate that WD-40 subdomains maintain their regulatory role in apoptosome even after Apaf-1 oligomerization [15].

In this study, we used a new split luciferase complementary assay for investigation of Apaf-1: Apaf-1 interactions in cell-based and cell-free systems [17,18]. Split luciferase complementary assay has been used to study apoptosome formation in differentiation of mouse embryonic stem cell to cardiomyocytes [19]. A similar strategy has been used to monitor of α-synuclein aggregation into amyloid fibrils, as a crucial factor leading to pathogenesis of Parkinson's disease (PD) [20]. Here in, Apaf-1 is fused through its N-terminus to either the N-terminal fragment (amino acids 1–416) or C-terminal fragment (amino acids 395–550) of *P. pyralis* luciferase. It is suggested that Apaf-1: Apaf-1 interactions bring Apaf-1 CARDs into close proximity which in turn allow N-luc and C-luc fragments to come in close proximity and reconstitute luciferase activity. It should be pointed out that apoptosome formation were studied in two different systems. In cell-based system, after induction of apoptosis, the apoptosome formation is assessed in extracts of the cells expressing Apaf-1 fused to complementary fragments of luciferase while in cell-free system, the apoptosome formation is induced in extracts of the cells that produce the aforementioned Apaf-1 by addition of dATP and cytochrome *c*. Most importantly, we found that full length Apaf-1 generated higher levels of luciferase activity in cell-based system compared to extracts of cell-free system. Furthermore, we have shown that truncated Apaf-1 lacking both WD-40 subdomains (ΔApaf-1) is able to interact with endogenous Apaf-1 and make apoptosome complexes which have a variety of molecular weights. Overexpression of ΔApaf-1 brought about with caspase-9 cleavage without caspase-3 activation.

## 2 Material and methods

### 2.1 Apaf-1 cloning

For cloning of Apaf-1 we used pcDNA3.1 vector. Apaf-1 sequence was PCR amplified using high fidelity PrimeSTAR GXL DNA Polymerase (Clontech) from FastBAC vector containing the His tagged Apaf-1 [21]. PCR-amplified N-terminal (1–416) and C-terminal (395–550) luciferase fragments were derived from pGL3 plasmid encoding *Photinus pyralis* luciferase and used for constructing split reporter [22,23]. A flexible Gly-Ser peptide linker was used to fuse either N-luc or C-luc luciferase fragment to Apaf-1. Binding of N-luc and C-luc fragment of luciferase to Apaf-1 was performed by in-fusion system. To construct the truncated mutant, a stop codon (amino acid 590) was introduced. Finally, nucleotide sequences of plasmids were confirmed by DNA sequencing.

### 2.2 Transfection and cell extract preparation

The human embryonic kidney cells (HEK 293) were transfected by the constructs using Polyethyleneimine (PEI) (1 mg mL^−1^) (1 μl of PEI per each μg of DNA). A day before transfection, 8 × 10^6^ cells were cultured in Dulbecco's modified eagle's medium (DMEM, high glucose; Sigma) supplemented with 10% fetal bovine serum (Sigma) and 1% penicillin/streptomycin (Sigma) solution in 150 mm diameter dishes and for each dish, 40 μg DNA was used for transfection. Cells were then incubated for 24 h at 37 °C, 5% CO_2_. Briefly, to supply S-100 extracts, the cells were then harvested by trypsinization and resuspension in extraction buffer (50 mM HEPES pH 7.0, 10 mM KCl, 5 mM EGTA, 2 mM MgCl_2_, 1 mM DTT plus cytochalasin B and protease inhibitors cocktail) [24]. Afterward, cells were lysed through three cycles of freezing and thawing in liquid nitrogen. Finally, the lysates were centrifuged at 100000 × g. Bradford assay carried out to determine the protein concentration of the lysates.

### 2.3 Luminescence assay in cell-based and cell-free systems

Before transfection, sterile high-quality DNA plasmids (N-luc Apaf-1 and C-luc Apaf-1) were prepared by midi-prep kit (Qiagen). For transfection, 22 × 10^4^ cells per well were cultured in 6-well plates and incubated at 37 °C, 5% CO_2_ for 24 h to reach 60–70% confluency. Starvation was carried out 4 h before transfection by incubating the cell with serum free DMEM. The mixtures of PEI and 2 μg of N-luc Apaf-1 and 2 μg of C-luc Apaf-1 constructs were prepared; (N/P 5, 12 and 20) and incubated at room temperature for 30 min. N/P ratio indicate the ratio of PEI (Nitrogen holder) to plasmid (Phosphorus holder) DNA. Finally, 50 μl of the transfecting complexes were added to each well and the cells were incubated for 4 h the medium were then replaced with 2 ml of DMEM supplemented with 10% FBS in each well and incubated for 24 h. Afterward, the culture media were replaced by the fresh ones and apoptosis was induced in the co-transfected cells (with N-luc Apaf-1 and C-luc Apaf-1 constructs) by different concentrations of doxorubicin (0.25, 0.5, 0.75 and 1 μM). Finally, the cell lysates were prepared 12, 24, 28 and 36 h after cell death induction and split-luciferase assays were carried out. The media were completely removed and 100 μl of CCLR (Cell Culture Lysis Reagent) buffer was added to each well on ice and incubated for 20 min. The lysates were then collected and 15 μl of each cell lysate was added to 15 μl of luciferase substrate cocktail. Results of co-expression of N-luc Apaf-1 and C-luc Apaf-1 constructs are a percentage of the highest luciferase activity.

In cell-free system to measure luciferase activity, 5 μl of each extract from N-luc Apaf-1 and C-luc Apaf-1expressing cells were mixed in each well of a 96 well plate. Afterward the following compounds were added in order: 2 μL cytochrome *c* (200 μg ml^−1^ stock solution), 3.5 μL dATP of a 10 mM stock solution (1.6 μM and 1.0 mM final concentration; respectively) and luciferase activity was immediately measured upon addition of 30 μL of luciferase substrate mixture including (D‐luciferin 5 mM, ATP 40 mM, MgSO_4_ 100 mM, Tris pH 7.8) using a Plate reader (PerkinElmer, England) at room temperature. Results are expressed as relative light units second^−1^ mg protein^−1^ (RLU s^−1^ mg^−1^).

### 2.4 Caspase-3 activity assay

Caspase-3 activity was only measured in cell free extracts. Before measuring caspase activity using DEVD-AMC [24], different proportions of untransfected and N-luc ΔApaf-1 transfected cell extracts were prepared and mixed in the presence and absence of cytochrome *c* (1.6 μM final concentration) and dATP (1 mM final concentration) for 15 min at 25 °C. Then each of the cell extract samples were mixed and the final reaction volume was adjusted to 200 μl with assay buffer containing HEPES 200 mM, DEVD-AMC 40 μM and DTT 5 mM. Finally, the observed fluorescent intensity was normalized to the protein concentration.

### 2.5 Gel filtration

Proteins in cell-free extracts of native and ΔApaf-1 (S-100 extracts) were prepared, mixed in presence and absence of dATP/Cc and then separated by gel filtration chromatography using a Sephacryl 300 column, as previously described [25].

### 2.6 Immunoblotting

Immunoblotting was carried out to assess the expression of recombinant proteins. To do this, 20 μg of protein sample from cell extract was resolved by gel electrophoresis and transferred onto a nitrocellulose membrane. The membrane was blocked by phosphate-buffered saline (1X PBS, pH 7.4) including 5% skimmed milk at room temperature for 2 h and then soaked in primary antibodies solution: anti-Apaf-1, mAb (AdipoGen, AG-20T-0134-c100), anti-caspase-3 (Cell Signaling Technology, 9662), beta Actin (Proteintech, 66009-1-Ig), or anti-caspase-9 (Cell Signaling Technology, 9502), all diluted 1:1000, and incubated at 4 °C overnight. The membrane was washed with PBS containing 0.05% Tween 20 (PBST) for 30 min before being incubated with secondary antibodies that were diluted 1: 10,000 (Goat anti-mouse; Li-cor, 926–68020), (Goat anti-rabbit; Li-cor, 926–32211), (Goat anti-rat; Li-cor, 925–32219) and (Goat anti-rabbit; Li-cor, 926–32211) in PBS. The membrane was scanned using a LI-COR system.

## 3 Results

### 3.1 Induction of apoptosis

**Fig. 1:**
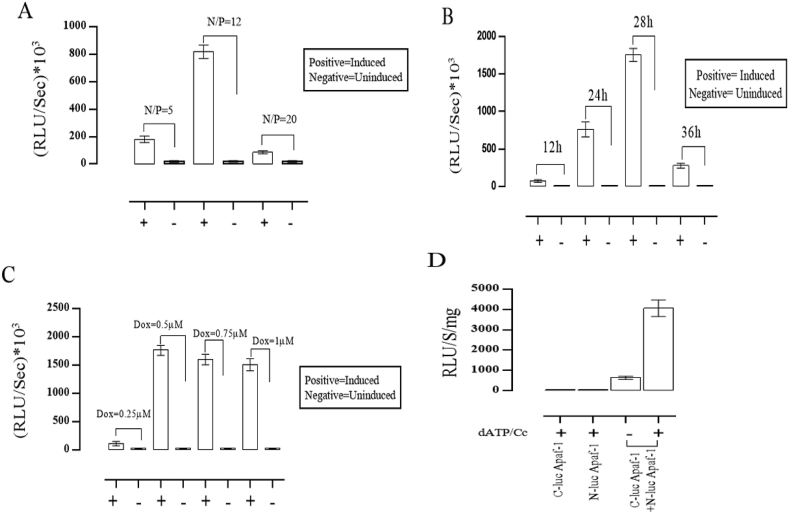
Reconstitution of luciferase activity upon Apaf-1 oligomerization in a cell-free and cell-based systems. (A) The effect of various N/P ratio on transfection efficiency. HEK293 cells were transiently transfected with 2 μg of established plasmids (C-luc Apaf-1 and N-luc Apaf-1) after 24 h with and without 0.5 μM doxorubicin. (B) Time-dependent apoptosis activation in (N/P 12) and 0.5 μM doxorubicin. (C) Apoptosis activation in the absence and presence of different concentrations of doxorubicin. Cells were co-transfected with 2 μg from C-luc and N-luc Apaf-1 (N/P 5) and after 24 h' luciferase activity was assessed. (D) Luciferase activity was evaluated in a mixture of C-luc Apaf-1 and N-luc Apaf-1 extracts with or without dATP and cytochrome *c*. Activity of C-luc Apaf-1 and N-luc Apaf-1 extracts alone were also tested. Results are the mean ± SD of three separate experiments.

We have previously reported split luciferase assay for monitoring apoptosome formation in living cells [26]. According to this report 0.5 μM concentration of doxorubicin (as a chemotrophic and apoptotic agent) was chosen as optimal concentration for induction of apoptosis [26]. In this study, in order to compare Apaf-1: Apaf-1 interaction in cell lysates and extracts of cell-free system, different N/P ratio, doxorubicin and time variables were tested to evaluate apoptosome formation based on complementation of luciferase fragments. To determine the optimal N/P ratio, as described in the experimental part, N-luc Apaf-1 and C-luc Apaf-1 co-transfection was carried out. As indicated in Fig. 1A, the highest luciferase complementary activity was observed at N/P 12, within 24 h after apoptosis induction by 0.5 μM of doxorubicin. The best time for apoptosis induction at N/P 12 and 0.5 μM of doxorubicin was obtained to be 28 h after cell death induction [Fig. 1B]. In the same manner, the highest luciferase activity at N/P 5 within 24 h and 0.5 μM of doxorubicin was obtained [Fig. 1C]. We detected a considerable fold change (∼10, 130 and 40 fold) in luciferase activity compared to uninduced cells at 12, 24 and 36 h after apoptosis induction, respectively. It reached a maximum luminescence signal over 28 h (∼280 fold), that represents increasing apoptosome complex (Apaf-1: Apaf-1 interactions) levels.

#### 3.1.1 Split reporter assay upon apoptosome formation

As previously reported, the recovery of luciferase activity occurs whenever two fragment of luciferase proteins bind each other [17]. However, split luciferase complementation assay was made while N-luc and C-luc fragments fused to the N-terminal of Apaf-1 proteins to assess Apaf-1: Apaf-1 interaction. Cell-free systems using extracts from eukaryote cells are increasingly being used to study apoptosome formation. According to the previous reports, adding exogenous dATP and cytochrome *c* (dATP/Cc) can trigger apoptosome formation [24]. Therefore, in cell-free system to prove the possibility of detecting Apaf-1: Apaf-1 interactions, N-luc and C-luc full length Apaf-1 extracts were first mixed, dATP/Cc was added and then split luciferase activity was measured. As indicated in Fig. 1D, in the presence of dATP/Cc a remarkable increase (∼5 fold) in reconstituted luciferase activity was observed compared to a mixture without cytochrome *c* and dATP. This results suggest that split reporter complementation assay was used for apoptosome formation in cell-free system as well even its fold induction is much lower than death induced in cell-based extracts.

### 3.2 ΔApaf-1 oligomerization by gel filtration

**Fig. 2:**
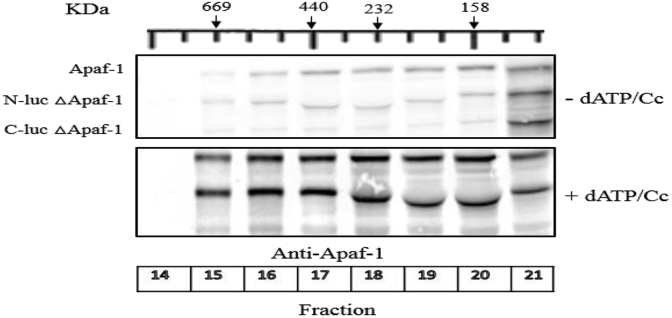
Gel filtration and immunoblotting analysis of ΔApaf-1. Extracts expressing N-luc ΔApaf-1 and C-luc ΔApaf-1 were mixed 1:1 (0.5 ml from each one) with or without cytochrome *c* and dATP and then incubated for 15 min at 25 °C and loaded on Sephacryl 300 HR column. The fractions collected, concentrated and detection of N-luc or C-luc ΔApaf-1 constructs was carried out using immunoblot.

For further characterization of apoptosome formation in cell-free system while Apaf-1 is linked to N-luc and C-luc fragments through a flexible linker, gel filtration chromatography was used before and after induction of apoptosome formation by dATP and cytochrome *c* for native and truncated Apaf-1 [Fig. 2]. To determine mini-apoptosome complex by ΔApaf-1, N-luc ΔApaf-1 and C-luc ΔApaf-1 cell extracts were prepared and then incubated with or without cytochrome *c* and dATP for 15 min at 25 °C. Gel filtration chromatography carried out using Sephacryl 300 HR column and the fractions were collected in the range of ∼700 kDa to 158 kDa (Fraction 14–21). Then, we detected the recombinant proteins by immunoblotting. As previous report, apoptosome complex has a characteristic molecular mass of ∼700 kDa. Therefore, gel filtration was performed to investigate whether N-luc and C-luc ΔApaf-1 take part in the formation of the accurate size protein complex [Fig. 2]. However, addition of cytochrome *c* and dATP, the monomers of N-luc and C-luc full lenght Apaf-1 shift to fraction with higher molecular weight fractions [data not shown] which indicated apoptosome formation, similar to native apoptosome [25]. While immunoblotting of truncated Apaf-1 (ΔApaf-1) indicates its oligomerization is independent of cytochrome *c* and this confirms the previous results regarding spontaneous oligomerization of truncated Apaf-1 lacking both WD40 domains. Interestingly, even in the absence of dATP/Cc the complexes with various size and form were observed which suggests that preformed complex has already existed due to the endogenous dATP and cytochrome *c* in the S-100 extract.

### 3.3 Elution of Apaf-1 oligomers from Ni-NTA affinity resin

**Fig. 3:**
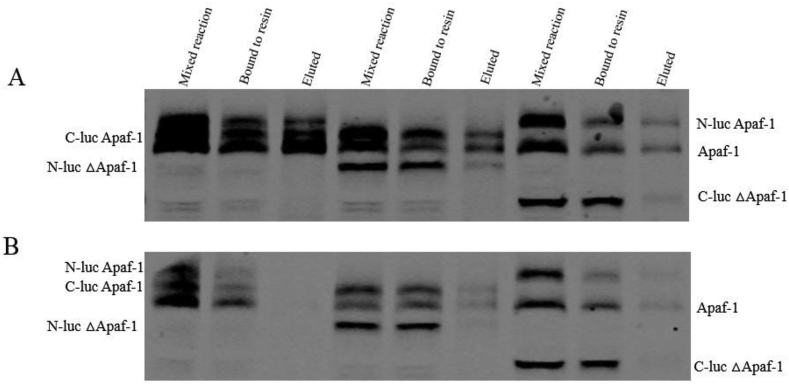
Binding of apoptosome complex to Ni-NTA Agarose Beads. Mixture of N-luc Apaf-1/C-luc Apaf-1; C-luc Apaf-1/N-luc ΔApaf-1 and N-luc Apaf-1/C-luc ΔApaf-1 were prepared, activated with or without cytochrome *c* and dATP for 15 min at 25 °C and then the mixtures incubated with resin for 1–2 h at 4 °C. Washing was performed in 20 mM imidazole (A) and 165 mM imidazole (B). Fractions were immunoblotted using anti-Apaf-1 monoclonal antibody.

Complexity in gel filtration eluate of ΔApaf-1 (compared to native form) may indicate different behavior in the endogenous Apaf-1 and ΔApaf-1 interaction. The presence of His-tag in the C-terminal of full length C-luc Apaf-1 construct was used for binding of apoptosome complex using (Ni-NTA) agarose affinity chromatography. As indicated in Fig. 3A and B a mixture of different Apaf-1 extracts was used in this experiment. Mixture of N-luc Apaf-1/C-luc Apaf-1; C-luc Apaf-1/N-luc ΔApaf-1 and N-luc Apaf-1/C-luc ΔApaf-1 were prepared, activated by cytochrome *c* and dATP. After application of mixture to the resin, they were washed by 20 mM and 165 mM imidazole. Simple mixture, bound and eluted fractions were analyzed by anti-Apaf-1 monoclonal antibody. As indicated in Fig. 3A and B, all 3 mixtures including N-luc Apaf-1/C-luc ΔApaf-1 complex (without any his-tag in the components) were bound to the resin. Washing of bound protein with 20 mM imidazole did not elute protein from the resin, while washing of resin with 165 mM imidazole eluted only N-luc Apaf-1/C-luc Apaf-1 mixture from the resin. Electrostatic interactions play a very important role to identify mechanisms of protein-protein complexation, thermostability, molecular identifications, structural adaptations, and protein motions [29]. Therefore, our results suggest that the interactions between full length Apaf-1 and ΔApaf-1 is stronger than full length Apaf-1: Apaf-1 interactions. It seems upon expression of ΔApaf-1 protein in the cells they constitutively formed stable apoptosome complexes through stronger interactions with other truncated Apaf-1 and endogenous Apaf-1.

### 3.4 Interaction between truncated Apaf-1 (ΔApaf-1) and endogenous Apaf-1

**Fig. 4:**
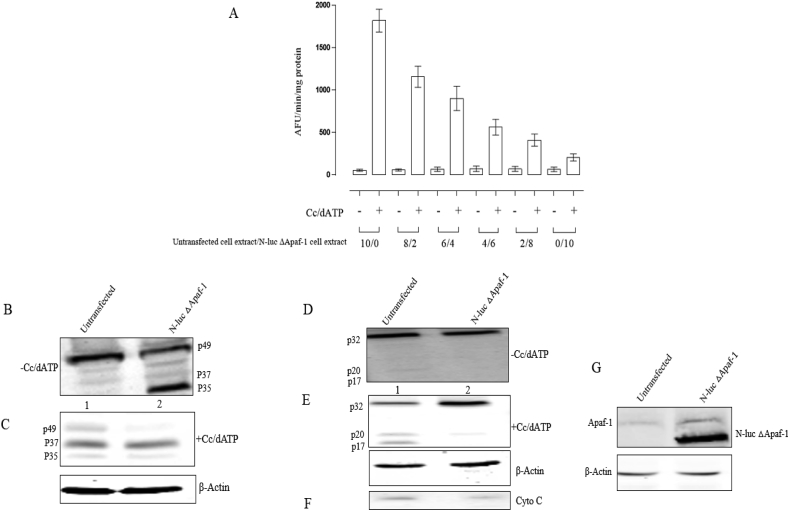
(A) Different proportions of untransfected and N-luc ΔApaf-1 were prepared, mixed and incubated in the absence and presence of cytochrome *c* and dATP for 15 min at 25 °C. Then caspase-3 like activity was detected using DEVDA-AMC. (B, C) Procaspase-9 processing without and with dATP/Cc, respectively. (D, E) Procaspase-3 processing in the absence and presence of dATP/Cc, respectively. (F, G) The level of cytochrome *c* and endogenous Apaf-1 expression in untransfected and N-luc ΔApaf-1 cell extracts, respectively.

As previous reported, ΔApaf-1 form a mini-apoptosome structure, cytochrome *c* independent that is able to activate caspase-9 but no caspase-3 [27]. To investigate the interaction between endogenous Apaf-1 and ΔApaf-1, cell extracts were prepared from untransfected and transfected cells with only N-luc ΔApaf-1 and mixed in different proportions. Then caspase-3 activity was measured in the absence and presence of dATP/Cc [Fig. 4A]. The expression level of cytochrome *c* [Fig. 4F] and endogenous Apaf-1 [Fig. 4G] in untransfected and ΔApaf-1 transfected cell extracts is the same, while the level of N-luc ΔApaf-1 protein was higher than endogenous Apaf-1 [Fig. 4G].

By increasing the amount of ΔApaf-1 cell extract, caspase-3 activity decreases that suggests the inhibitory role of ΔApaf-1 by interacting with endogenous Apaf-1 and reducing caspase-3 activity. To further assess reducing role of N-luc ΔApaf-1, the processing of procaspase-9 and procaspase-3 were performed. Comparison of procaspase-9 processing from untransfected and ΔApaf-1 cell extracts in the absence of cytochrome *c* and dATP showed that procaspase-9 processing level from N-luc ΔApaf-1 cell extract is much higher than untransfected cells [Fig. 4B, lane 1 and 2]. While the presence of dATP/Cc enhances procaspase-9 processing level from untransfected cell extract and a similar level of procaspase-9 processing in both cell extracts was observed [Fig. 4C, lane 1 and 2].

On the other hand, a low level of caspase-3 processing from untransfected cells was not observed in N-luc ΔApaf-1 cell extract [Fig. 4D, lane 1 and 2] but when cytochrome *c* and dATP were added to the both extracts, a significant increase in caspase-3 processing level has been observed in untransfected cell extracts compared to N-luc ΔApaf-1 cell extract in which no processing has been observed [Fig. 4E, lane 1 and 2].

## 4 Discussion

Apaf-1 is a multi-domain protein including caspase recruitment domain (CARD), a central nucleotide-binding domain (NOD) and C-terminus multiple WD-40 repeats that has a regulatory function [[7], [8], [9], [10]]. When cytochrome *c* binds to the WD-40 repeats [9] making structural changes in Apaf-1, leading to exposure of nucleotide binding sites to dATP/ATP, oligomerization occur and Apaf-1 assembles into a wheel like apoptosome complex [2,7]. Split-luciferase complementary assay has been used to show apoptosome formation in the cells upon apoptosis induction [26]. As indicated in Fig. 1, co-expression of N-luc Apaf-1/C-luc Apaf-1 in the cells followed by apoptosis induction brought about with a significant fold induction (280 fold induction in optimum condition) in luciferase complementary activity in cell extracts of induced cells (cell-based system) compared to uninduced cells, while separate expression of N-luc Apaf-1 and C-luc Apaf-1 followed by extract preparation and induction of apoptosome complex by adding of dATP/Cc brought about with only 5 times fold induction in luciferase activity (cell-free system). Therefore, it may simply have suggested that higher luciferase activity may suggest other required factors for proper Apaf-1 oligomerization and apoptosome formation in the cells while they were missed in cell-free system for apoptosome formation.

Moreover, preparation of N-luc Apaf-1/C-luc Apaf-1 constructs enabled us to see the effect of truncated mutant of Apaf-1 (ΔApaf-1) on apoptosome formation in cell-free system. Introduction of a stop codon at the end of NOD domain (residue 590) produced truncated mutant of N-luc Apaf-1 and C-luc Apaf-1; N-luc ΔApaf-1 and C-luc ΔApaf-1, respectively. For further characterization of apoptosome formation in cell-free system while Apaf-1 is linked to N-luc and C-luc fragments through a flexible linker, gel filtration chromatography was used before and after induction of apoptosome formation by dATP and cytochrome *c* for native and truncated Apaf-1 [Fig. 2]. Our study demonstrated that the lack of both WD-40 subdomains of Apaf-1 produced an autoactivated variant (ΔApaf-1) which can automatically oligomerize and trigger activation of procaspase-9 independent of dATP/Cc, as reported earlier [24,27]. According to this idea, oligomerization process plays a crucial role in activating procaspase-9. On the other hand, cytochrome *c* and dATP have a regulatory role in the full length Apaf-1 oligomerization process, it seems that deletion of both WD-40 subdomains can be effective on oligomerization process. It has been reported, upon addition of dATP/Cc to cell lysate, two large ∼1.4-MDa and ∼700-kDa apoptosome complexes are formed [28]. Gel filtration was performed to investigate whether N-luc and C-luc ΔApaf-1 take part in the formation of the accurate size protein complex [Fig. 2]. It should be noted, upon addition of cytochrome *c* and dATP, the monomers of N-luc and C-luc full length Apaf-1 shift to fraction with higher molecular weight fractions [data not shown] which indicated apoptosome formation, as reported earlier [28]. While immunoblotting of truncated Apaf-1 (ΔApaf-1) indicates its oligomerization is independent of cytochrome *c* and this confirms the previous results regarding spontaneous oligomerization of truncated Apaf-1 lacking both WD-40 subdomains. Interestingly, even in the absence of dATP/Cc the complexes with various size and form were observed which suggests that preformed complex has already existed due to the endogenous dATP and cytochrome *c* in the S-100 extract. It seems that presence of ΔApaf-1 can form apoptosome complexes with a range of complexity through interaction with endogenous Apaf-1. Moreover, while endogenous Apaf-1 and N-luc or C-luc truncated Apaf-1 have not been observed in fraction 14 and lower, indicates lack of ∼1.4-MDa apoptosome complex [Fig. 2].

On the other hand, binding of N-luc Apaf-1/C-luc Apaf-1; C-luc Apaf-1/N-luc ΔApaf-1 and N-luc Apaf-1/C-luc ΔApaf-1 mixtures to the affinity column were performed [Fig. 3]. It should be noted, binding of N-luc Apaf-1/C-luc ΔApaf-1 mixtures (without His-tag) to the resin indicate role of non-specific interactions in binding. Washing of bound proteins with 20 mM imidazole did not elute protein from the resin, while washing of resin with 165 mM imidazole eluted only N-luc Apaf-1/C-luc Apaf-1 mixture from the resin. It may be concluded, while mixture of N-luc Apaf-1/C-luc Apaf-1 has only one His-tag, its binding takes place through specific interaction. Elution of C-luc Apaf-1 by 165 mM imidazole brought about with elution of N-luc Apaf-1 which for C-luc Apaf-1/N-luc ΔApaf-1 mixture in spite of presence of one His-tag in C-luc Apaf-1 was not happened. Therefore, it may be suggested only in mixture of N-luc Apaf-1/C-luc Apaf-1 right “juxtaposition” of CARD-domain of Apaf-1 molecules formed a real apoptosome structure which could bind to the resin. While in the other mixture (C-luc Apaf-1/N-luc ΔApaf-1) in spite of intact CARD-domain geometry of Apaf-1oligomeres His-tag is not in right orientation for binding to Ni-NTA resin and they were bound through non-specific (electrostatic) interaction. However, we could not rule out the possibility that CARDs of truncated Apaf-1 might have been disordered when exposed to full length Apaf-1. To test this idea, we showed that N-luc Apaf-1 (without His-tag) is released from apoptosomes assembled with C-luc Apaf-1 (with His-tag), when they were eluted with 165 mM imidazole. Lack of eluted protein even at high imidazole concentration for truncated mutant (ΔApaf-1) indicated predominant role of non-specific interaction in their binding.

Role of truncated mutant of Apaf-1 (ΔApaf-1) overexpression on activation of caspase-9 and caspase-3 indicates a dominant negative effect for mutant Apaf-1 (ΔApaf-1) on endogenous Apaf-1 [Fig. 4]. Autoprocessing of caspase-9 without caspase-3 activation upon overexpression of ΔApaf-1 is similar to the results of a mini-apoptosome structure from an Apaf-1 without WD-40 repeats [[30], [31], [32]].

In conclusion, according to the results in this manuscript, it may be concluded that preparation of a luciferase complementary assay for apoptosome formation may shed light on additional required constituents in apoptosome formation and effective ionic bond [33] in latent form of Apaf-1.

## Conflicts of interest

The authors report no conflict of interests.
